# Characterization, stability, and feasibility of long-term use of light-absorbing components of aqueous spinach extract-based photogalvanic electrolyte

**DOI:** 10.1038/s41598-022-17647-5

**Published:** 2022-08-07

**Authors:** Pooran Koli

**Affiliations:** Department of Chemistry, Jai Narain Vyas University, Jodhpur, Rajasthan 342033 India

**Keywords:** Chemistry, Energy science and technology, Materials science, Optics and photonics

## Abstract

In the present work, the photogalvanic cells have been studied with respect to the photo-stability and the long-term use of the electrolyte based on crude aqueous spinach extract sensitizer for solar energy harvesting. Further, the nature of chemical components present in the old and photo-decayed electrolyte and their current generation capacity has also not been investigated so far otherwise it is of much significance for durable use of the same electrolyte in cells. In earlier studies, the steady-state photo-generation of current for about two hours from crude spinach extract-based cell has been shown during illumination. But, the data for only two hours of the steady-state current generation is not sufficient to show the feasibility of working with photogalvanic cells. Therefore, to fill this research gap of lack of characterization of sensitizers’ molecules of crude spinach extract and lack of study on long-term use of this electrolyte (crude spinach extract-surfactant-reductant-alkali-water), the present extensive study has been done. The observed spectrum of crude spinach extract resembles that of chlorophyll–protein complex showing it is the main chemical component in extract absorbing light. A strong acid adversely affects the extract’s photogalvanics and high pH is friendly to the physiological and photogalvanic activity of the extract. The spectra of illuminated and very old crude spinach extract-NaOH-Sodium lauryl sulfate (NaLS)-Fructose photogalvanic electrolyte solution show negligible absorbance (540–700 nm) and zero absorbance (at 700 nm) suggesting the absence of chlorophyll due to its photo-degradation. When this photo-degraded electrolyte is again illuminated, the power output obtained is nearly equal to that for the first time illuminated fresh electrolyte. The observed current at zero time and after 2641 h from the same electrolyte used in long term is 50 mA cm^−2^ and 40 mA cm^−2^, respectively. It means that the fresh crude spinach extract, as well as the photo-degraded extract at high pH, are almost equally capable of power generation.

## Introduction

A photogalvanic cell device converts solar energy into solar power. A photo-galvanic cell consists of the two electrodes dipped in the photo-sensitizer and reductant based electrolyte solution^1–4^. All promising solar cell devices^5–7^ including the photo-galvanic cells have to be sustainable and durable in use. The sustainability and durability of the photogalvanic cells holds special significance as photo-sensitizer molecules are prone to photo-decay. The characterisation, stability, and feasibility of long-term use of light absorbing chemical components of crude aqueous spinach extract electrolyte is of paramount importance, but the same has not been scientifically investigated in photogalvanics. Photogalvanicists have mainly focussed on use of the artificial/synthetic dye photo-sensitizers, synthetic reductants, synthetic surfactants and synthetic alkali/buffer solution to constitute the electrolyte for the solar energy harvesting through the photogalvanic cells. Some of the synthetic sensitizer-synthetic reductant chemical systems reported are as Thionine dye sensitizer-Iron reductant^8^, Rhodamine B dye-Fructose reductant^9^, Sudan-I dye-Fructose reductant^10^, Bromophenol Red dye–EDTA reductant^11^, Safranine O-EDTA reductant^12^, Toluidine Blue dye-glucose reductant^13^, Toluidine Blue dye-Fe(II)reductant^14^, and Congo red dye-Formaldehyde reductant^15^.

The use of synthetic dye sensitizers is against the ultimate aim of the sustainability and renewable nature of the solar energy harvesting. Further, the characterisation of photo-decay products of dye sensitizer molecules constituting the electrolyte and their significance in current generation through cell has also evaded the attention of the researchers. Although, some studies on the use of sustainable, renewable, and natural resources (like spinach extract) for solar energy harvesting through photogalvanic cells have been reported^16,17^. Photogalvanic cells based on crude aqueous spinach extract sensitizer (a sustainable, renewable, and natural resource)-Sodium Lauryl Sulfate (NaLS) surfactant-Fructose reductant-NaOH alkali electrolyte have been studied and reported for solar energy conversion and storage at low^16^ and high illumination intensity^17^. But, the characterization of light absorbing materials present in the crude aqueous extract of spinach has not been done in these studies. Further, the nature of chemical components present in the old and photo-decayed electrolyte and their current generation capacity has also not been investigated otherwise it is of much significance for durable use of same electrolyte in cell. Although, steady-state photo-generation of current for about two hours from crude spinach extract based cell has been shown during illumination. But, the data for only two hours steady-state current generation is not suffice to show the feasibility of working of photogalvanic cells. Therefore, to fill this research gap of lack of characterisation of sensitizers’ molecules of crude spinach extract and lack of study on long-term use of this electrolyte (crude spinach extract-surfactant-reductant-alkali-water), the present extensive study has been done.

In present study, the sensitizer present in the crude aqueous spinach extract has been characterised by the UV–visible spectroscopic study. Further, the changes occurring in the spectral property of that sensitizer under the influence of NaOH alkali, HCl acid, NaLS surfactant, fructose reductant, photo-illumination, and time duration has also been studied spectroscopic ally. The suitability of high pH and un-suitability of low pH for better solar cell efficiency has also demonstrated through an insight into change in electrolyte. The feasibility of long-term use of same electrolyte of crude spinach extract for solar energy harvesting has also been demonstrated for over 2500 h.

## Materials and methods

The present study has used crude aqueous extract of the spinach leaves along with the Fructose (99.8% Assay-Purity), Sodium lauryl sulfate-NaLS (94% minimum Assay-Purity), and NaOH (98% Assay-Purity). The stock solutions of all the chemicals have been prepared by direct weighing and dissolving in the single distilled water, and solutions have been kept in dark to protect them from light^16,17^.

Authors have prepared the fresh extract using spinach leaves. Spinach leaves has been used as source of the natural photo-sensitizer by author as spinach leaves has some specific features vis-à-vis other green leaves. Some of the special features of the spinach are as- (i) fresh spinach is a convenient source of chlorophyll ‘a’ & ‘b’. chlorophyll ‘a’ is the dominating light harvesting material among all the pigments found in the crude aqueous extract of the spinach leaf; (ii) spinach is identified to be most rich in chlorophyll. The study on chlorophyll contents of some raw vegetables has reported that raw spinach has highest chlorophyll content (mg kg^−1^ fresh weight)^18^, i.e., of the order of 1270. The reported chlorophyll contents (mg kg^−1^ fresh weight) of Green beans, Brussels sprouts, Broccoli, Parsley, Cucumbers, Green peas, Leeks, Green paprika, Zucchini, Celery is of the order of 71–133, 60, 128, 995, 36, 50, 87, 38, 68, and 34, respectively^18,19^; (iii) spinach extract containing natural dye is a common natural sensitizer. The dye extracted from spinach is a proven robust natural dye in DSSC studies^20^; and (iv) spinach is a bio-material which is widely available throughout the year.

The natural photo-sensitizers present in the spinach extract have not been separated for use, but extract as such has been used. Aqueous Spinach Extract has been obtained through procedure as (i) first of all, the fresh spinach leaves are washed with water, (ii) the washed and wet leaves (50 g) are crushed in the presence of a little water (10 ml) in a Jar of electric mixer, (iii) then crushed matter is filtered with a ordinary filter paper, (iv) filtrate is left undisturbed for some time to allow sedimentation of fibrous and other matter, and (v) the transparent liquid above sediment is used as aqueous Spinach Extract for photosensitization of solution in photogalvanic cell^16,17^.

Crude spinach extract as natural photo-sensitizer has also been widely used in the Dye-Sensitized Solar Cells (DSSC). But, the efficiency from the crude chlorophyll extract based DSSCs cell is very low owing to the failure of chlorophyll ‘a’ to bind with the semiconductor electrode. But, the NaOH hydrolyzed crude chlorophyll extract based DSSCs cells shows enhanced efficiency^21^. On hydrolysis of the crude chlorophyll extract, the ester linkage of the chlorophyll molecules undergoes chemical change to form –COOH and –OH groups which helps it to bind with the semiconductor electrode leading to the enhanced efficiency. As far as the photogalvanics of the crude chlorophyll extract is concerned, the anchoring of the chlorophyll molecule with the working electrode is not required. Further, the photogalvanic cells use NaOH. All these factors make chlorophyll even more efficient for light harvesting through the photogalvanic cells.

UV–visible spectra has been taken manually by a single beam UV–visible spectrophotometer-108 (Systronics, Ahmedabad, India). Cuvette cells used (Optiglass Ltd., UK) have transmissions 82.3% (at 200 nm), 84.3% (at 220 nm), and 85% (at 85 nm). From this spectrophotometer, the accuracy of band positions in the observed is ± 0.5 nm. Samples have been scanned manually by noting % transmittance (absorbance) at different wavelengths. UV–visible spectra has been drawn manually between absorbance (at ‘*Y*’ axis) and absorbed wavelength in nm (at ‘*X*’ axis). Fresh spinach is convenient source of the Chlorophyll a and b. The absorption band of the chlorophyll ‘*a*’ is at 660 nm (in red region) and 430 nm (in blue) in diethyl ether. The absorption band of chlorophyll-*b* is at 650 nm (in red region) and 453 nm (in blue) in diethyl ether. Under the present experimental conditions, the absorption bands of crude spinach extract have been observed at 680 nm (in red region) and 440 nm (in blue). Chlorophyll absorbs radiation almost over the range particularly in the blue-violet (470 nm) and red (650–700 nm)^16,17^.

The reproducibility of the phenomenon described in the manuscript has been checked in two ways-first, by comparing the spectral results of the present study with already published work, and, second, by repeating the observations of the electrical output of the same electrolyte by recharging at different time.

Author declares that the present experimental research has used an edible plant material (spinach leave available in open market). Present experimental research plant material has complied with relevant institutional, national, and international guidelines and legislation.

## Results and discussion

### UV–visible spectra of crude aqueous spinach extract and characterisation of extracts’ components responsible for light absorption in photogalvanic cell for solar power and storage

The spectra of crude aqueous spinach extract have been found similar to that for intact chloroplast, except in the UV region. The spectra of intact chloroplast have two absorption maxima at 678.5 nm (Q band; A_0.875_) and 437 nm (Soret band with A_1.8_)^22–24^.

**Figure 1:**
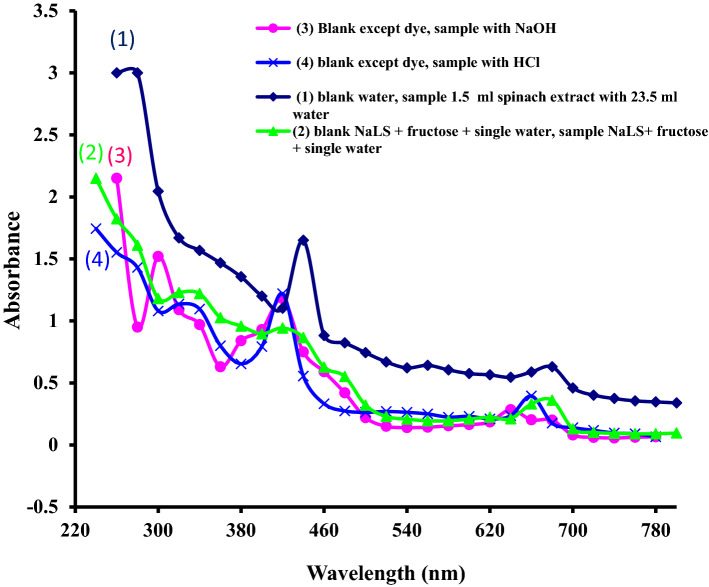
(1) UV–visible spectra of pure aqueous spinach extract^16^ [curve line 1,  Main spectral property: Absorbance (A) continuously increasing towards shorter wavelengths, bands at 680 nm (A_0.63_) and 440 nm (A_1.65_)]; (2) UV–visible spectra showing the effect of NaLS and Fructose on the spectra of aqueous spinach extract [curve line 2,  Main spectral property: Absorbance slightly but continuously increasing towards shorter wavelength, bands at 680 nm (A_0.36_), 420 nm (A_0.94_), 340 nm (A_1.22_), shoulder band at 480 nm (A_0.55_)]; (3) UV–visible spectra showing the effect of alkali NaOH on the spectra of spinach extract having NaLS and Fructose [curve line 3, Main spectral property: Absorbance slightly but continuously increasing towards the shorter wavelength, bands at 680 nm (A_0.36_), 420 nm (A_0.94_), 340 nm (A_1.22_), shoulder band at 480 nm (A_0.55_)]; and (4) UV–visible spectra showing the effect of HCl on spectra of spinach extract having NaLS and Fructose [curve line 4,  Main spectral property: Absorbance slightly but continuously increasing towards shorter λ, bands at 660 nm (A_0.39_), 420 nm (A_1.22_), 320 nm (A_1.13_), and shoulder band at 280 nm (A_1.43_)].

The UV–visible spectrum of crude spinach extract shows two main absorption bands at 680 nm (A_0.63_) and 440 nm (A_1.65_) in visible region with a slight, but continuous increase in absorbance towards shorter wavelengths, and strong absorption without any maxima in UV region^16^ (Fig. 1, curve line 1; Supplementary Fig. S1; Supplementary Table S1). The absorption spectrum of the crude aqueous spinach extract, which contains mixture of the *chlorophyll ‘a’* and ‘*b’* and carotenoids (see Sect. 1 of supplementary section-SI)*,* is dominated in the visible region by the absorption of *chlorophyll ‘a’* (allowed π → π* transitions). The *chlorophyll ‘b’* and the carotenoids absorb broadly in the blue region (400–500 nm). The band at 440 nm may be the resultant not only of *chlorophylls ‘a’ and ‘b’* but of the carotenoids as well^25^.

The absorption of crude spinach extract is slightly different than that for pure chlorophylls. The absorption maxima for pure chlorophyll ‘a’ is reported at 660 nm and 430 nm with nearly 1.3 intensity ratio (A_430_/A_660_) in ether solvent, and absorption maxima for pure chlorophyll ‘b’ in ether solvent is reported at 650 nm and 453 nm with nearly 3–4 intensity ratio (A_453_/A_650_) showing 650 nm band of chlorophyll ‘b’ is very weak^26^.

Lin and Shi has also reported a saddle-like spectrum with two peaks at 415 nm (Soret or ‘B’ band) and 664 nm (Q band) for chlorophyll^27^. The spectra of chlorophylls has been explained with the help of *Huckel's* rule, π–π^*^ transitions, and *Gouterman model* (four-orbital model). The chlorophyll’s porphyrin ring is an aromatic ring structure with 18 π electrons arranged in conjugated fashion. The *Gouterman model* attributes origin of Soret band to π–π^*^ strong electronic transition to the second excited state, and origin of Q band to π–π^*^ weak electronic transition to the second excited state. Four-orbital model has been proposed to explain the UV–visible absorption spectra of the porphyrin rings through the electron transitions from highest occupied molecular orbital (HOMO) to lowest unoccupied molecular orbital (LUMO). The nature of the central metal ions and the substituent’s constituting this ring affects the energy difference between the HOMO and LUMO and in turn its absorption spectra^28^. The position and intensity of absorption in visible region for crude spinach extract differs from pure chlorophylls mainly in two ways:

*First,* the bands of crude spinach extract are red shifted due to (a) high polarity of medium, and (b) pigment-chloroplast protein interactions as chlorophylls are associated with chloroplast protein and carotenoids through true chemical linkage and loose linkage, respectively^25,29^. The chlorophyll is neither free in crude aqueous spinach extract nor it can be as pure chlorophyll is insoluble in aqueous medium.

*Second,* the intensity of absorption of crude spinach extract is higher (with a slight but continuous increase towards shorter wavelengths) than that for pure chlorophyll.

It may be due to turbidity and suspended matter present in the crude spinach extract as the chlorophyll–protein complex of the spinach leaf is not in true solution (it is opalescent). The turbidity and light scattering leads to the higher absorbance between 400 and 800 nm with a slight, but continuous increase in the absorbance towards shorter wavelength. Turbidity leads to higher absorbance for the pigments. Turbidity can be checked by measuring *absorbance at* 750 nm and 520 nm. For a fully transparent leaf pigment extract, *absorbance at* 750 should equal zero, since chlorophyll ‘a’ (350–700 nm), and chlorophyll ‘b’ and carotenoids do not absorb in this region^25^. In present study, absorption at 750 nm and above is suggestive of presence of turbidity in crude spinach extract.

One more absorption band in visible region at 560 nm (A_0.64_) was also observed for crude spinach extract (Fig. 1, curve line 1). This band may be due to chlorophyll ‘a’- protein complex (one minor band near 560 nm also reported for chlorophyll ‘a’) reinforced by absorption due to other chemicals like Vt. B_2_ (λ_max_ near 565 nm) and B_12_ (one band near 550 nm)] present in crude spinach extract.

Chlorophylls are not efficient UV-absorbers but are still able to absorb UV radiation, especially around 350 nm^30^. No absorption below 350 nm is reported for chlorophylls. The strong absorption in UV region [260 nm (A_3_), 280 nm (A_3_), 300 nm (A_2.04_), 320 nm (A_1.67_), 340 nm (A_1.56_)] may be due to allowed π → π* absorption by chemicals (Vitamins like A_1_, A_2_, B_1_, B_12_, E; other than Vt.B2; alkaloids like niacin; acids like oxalic acid, omega-3 fatty acids, p-Coumaric acid; proteins, etc.) present in crude spinach extract (Sect. 1 of SI). Authors’ view is based on the fact that the λ_max_ for these chemicals lies in UV region.

The electronic absorption spectrum of the crude spinach extract is dominated by the chlorophyll ‘a’ type absorbance. This spectrum of crude extract has extreme resemblance to the spectra of chlorophyll–protein complex (intact chloroplast) (Fig. 1, curve line 1). Therefore, it may be inferred that chlorophyll–protein complex chromophore is the main chemical in the electrolyte (having the crude spinach extract) accounting for light absorption through photo-excitation in the visible region. Some other chemical components [e.g.,* carotenoids* (*λ*_*max*_* near 450 nm*),* Vt.B2, Vt.B12*] of the electrolyte (having the crude spinach extract) may also be playing role in the light absorption augmenting the absorption of the visible light by the chlorophyll–protein complex chromophore^17^ (Sect. 2 of SI).

In the crude spinach extract, the chlorophyll ‘b’ and carotenoids including other accessory pigments are thought to transfer absorbed energy to the *chlorophyll ‘a’ molecule*^31–33^. The chemicals like carotenoids and phycobilins are reported to sensitize the photosynthesis^34^. The chlorophyll–protein complex present in the crude extract is the main component harvesting the light. Chlorophyll may also act as electron donor via its ring E and keto groups present in ring and side chains^35^**.**

### UV–visible spectroscopic study of effect of surfactant NaLS and reductant Fructose on the crude spinach extract

In the presence of NaLS and Fructose, the crude spinach extract shows absorption at 680 nm (Q band with A_0.36_), 420 nm (Soret band with A_0.94_), 340 nm (A_1.22_), and one shoulder at band 480 nm (A_0.55_), with a slight but continuous increase towards shorter wavelengths (Fig. 1, curve line 2; Supplementary Fig. S1; Supplementary Table S1, Sect. 3 of SI). There is a hypo-chromic shift for absorbance at Q-band, Soret band, and for all wavelengths in comparison to pure crude spinach extract (Fig. 1, curve line 1). The pure aqueous NaLS solution alone shows absorption in UV region at λ_max_ 260 nm (A_max_ 0.04). NaLS causes blue shifts of 280 nm band (generally observed for each protein and amino acids) owing to conformational changes by disruption of hydrogen bonds. This change in absorption may be due to various reasons^25,29,36–38^ like NaLS induced removal of strong chlorophyll–chlorophyll interaction and pheophytinization coupled with conformational changes in the protein part of the chlorophyll complex (Sect. 4 of SI).

Reductants are reported to favor solubility and stability of proteins by disfavouring their denaturation^39^. It is reported that if there are molecules present that can act as oxidant or reductant, the energy of excitation may be expended in transferring an electron by oxidation/reduction of chlorophyll as below^24^:

$${\text{Chl}}^{*} \, + {\text{ Ox}} \to {\text{Chl}}^{. + } + {\text{ Ox}}^{. - } ; {\text{Chl}}^{*} \, + {\text{Red}} \to {\text{Chl}}^{. - } + {\text{Red}}^{. + } ,$$

$${\text{Chl}} + {\text{ Ox}} \to {\text{Chl}}^{. + } + {\text{ Ox}}^{. - } ;^{{}} {\text{Chl}} + {\text{Red}} \to {\text{Chl}}^{. - } + {\text{Red}}^{. + } .$$

The reactions are much faster if not coupled with proton transfer. Another possible process is electron ejection as^40^, Chl + light → Chl^.+^  + e^−^.

The inhibition to re-oxidation of reduced chlorophyll by the carotenoid, tetracene and anthracene^41^ is reported.

### UV–visible spectroscopic study of effect of the alkali NaOH on crude spinach extracts in the presence of surfactant NaLS and reductant Fructose

In the presence of NaOH (pH 13.71 with NaLS and Fructose), the crude spinach extract shows absorption at 680 nm (A_0.20_), 640 nm (A_0.28_), 420 nm (Soret band, A_1.18_), 300 nm (A_1.52_), and one shoulder at band 340 nm (A_0.97_), with a slight but continuous increase towards shorter wavelengths (Fig. 1, curve line 3; Supplementary Fig. S1; Supplementary Table S1).

There is a hyper-chromic shifted Soret band, a hyper-chromic and hypso-chromic shifted band at 300 nm, and a hypo-chromic shifted Q-band at 680 nm with one additional band at 640 nm in comparison to the spectra of crude spinach extract containing NaLS with fructose (Fig. 3, curve line 2). There is a hypo-chromic and hypso-chromic shifted Soret band, a hypo-chromic shifted Q-band at 680 nm with one additional band at 640 nm in Q-band region, an additional band at 300 nm and an additional shoulder band at 340 nm (A_0.97_) in comparison to the spectra of pure crude spinach extract (Fig. 1, curve line 1). In comparison to the spectra of pure crude spinach extract, the bands in NaOH are broad and less intense. This change in absorption spectrum may be due to saponifying^25^, solubilizing^25,42^, denaturizing^25^, and enolization^43–45^ effect of NaOH on chlorophyll–protein complex (Sect. 5 of SI).

In denaturation, it is to be mentioned that there is change in only secondary and tertiary structure of protein. Primary structure and porphyrin structure is reported to be intact^25^. It is reported that the hydrolysis of both chlorophylls with cold dilute alkali (KOH) solution gives one molecule of phytol, one molecule of methanol, and one molecule of chlorophyllide ‘a’ or chlorophyllide ‘b’^46^.

The spectrum of chlorophyll ‘a’ in the absence of NaOH has higher absorbance and sharp peaks. The profound alteration in the spectrum on making the phase test intermediate suggests that its negative charge is not confined to the oxygen of the enolate ion but is distributed over the entire conjugated system^24,43^. In this structure, the conjugated system no longer makes a closed loop, and an altered spectrum might be expected^47^.

### UV–visible spectroscopic study of effect of the HCl on crude spinach extracts in the presence of surfactant NaLS and reductant Fructose

In the presence of HCl (pH 0.29 with NaLS and Fructose), the crude spinach extract shows absorption at 660 nm (Q band, A_0.39_), 420 nm (Soret band, A_1.22_), 320 nm (A_1.13_), and one shoulder band near 280 nm (A_1.43_), with a slight but continuous increase towards shorter wavelengths (Fig. 1, curve line 4; Supplementary Fig. S1; Supplementary Table S1).

There is a hypo-chromic and hypso-chromic shifted Soret band at 420 nm, a hypo-chromic and hypso-chromic shifted Q-band at 660 nm, an additional band at 320 nm and a shoulder band near 280 nm (A_1.43_) in comparison to spectra of pure crude spinach extract (Fig. 1, curve line 1).

There is a hyper-chromic shifted Soret band, a hypo-chromic and hypso-chromic shifted band at 320 nm, and a hyper-chromic and hypso-chromic shifted Q-band at 660 nm with one additional shoulder near 280 nm in comparison to spectra of crude spinach extract containing NaLS with fructose (Fig. 1, curve line 2). This change in absorption spectrum may be due to pheophytinization, denaturization, and hydrolysis of chlorophyll–protein complex in acidic medium (Sect. 6 of SI).

### Effect of illumination on absorption spectra of crude spinach extract

**Figure 2:**
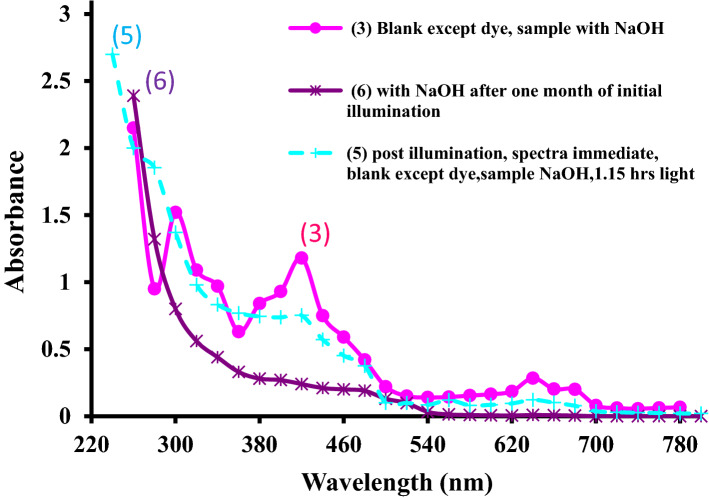
Effect of the illumination on the spectra of extract in the presence of NaOH, NaLS, and Fructose [curve line 5,  main spectral property: a spectrum was taken immediately after illumination. Absorbance slightly, but continuously increasing towards the shorter λ, broad band at 420 nm (A_0.75_), very broad band 640 nm (A_0.122_), shoulder band at 480 nm (A_0.37_)].

A UV–visible spectrum taken immediately after first time illumination of the photogalvanic solution is nearly similar to pre-illumination spectra in alkaline medium. The post-illumination spectra of crude spinach extract in alkaline medium in presence of NaLS and fructose contains hypo-chromic shifted broad bands in visible region (Fig. 2, curve line 5) in comparison to the pre-illumination spectra of same photogalvanic solution (Fig. 1, curve line 2). In UV region, the pre and post-illumination spectra for crude spinach extract is nearly same except at 260 nm and 280 nm.

After illumination, the absorption bands becomes broader and less intense in comparison to the bands of pure crude spinach extract (Fig. 1, curve line 1) as well extract with NaOH (Fig. 1, curve line 1) due to photo-bleaching of the chlorophyll and photon induced changes in protein moiety^48,49^. A post-illumination spectrum of crude spinach extract having NaOH, NaLS and fructose is only slightly different than that for pre-illumination spectra of same photogalvanic solution (Sect. 7 of SI). Reasons may be stability of chlorophyll to high light intensities for long periods in the aqueous extracts^25^, no-bleaching of chlorophylls in absence of organic solvents^50^, stable reduced semiquinones of the pigments^51,52^, reversibility as re-oxidation of reduced chlorophyll^53–63^.

### Post-illumination spectra of crude spinach extract in alkaline medium in presence of NaLS and fructose (spectra of 33 days old illuminated electrolyte)

Post-illumination spectra of crude spinach extract in alkaline medium in presence of NaLS and fructose was nearly similar to pre-illumination spectra of the same solution. This already illuminated photogalvanic electrolyte solution was again illuminated on 24th day of preparation of electrolyte, and then its UV–visible spectra were taken on 33rd day.

**Figure 3:**
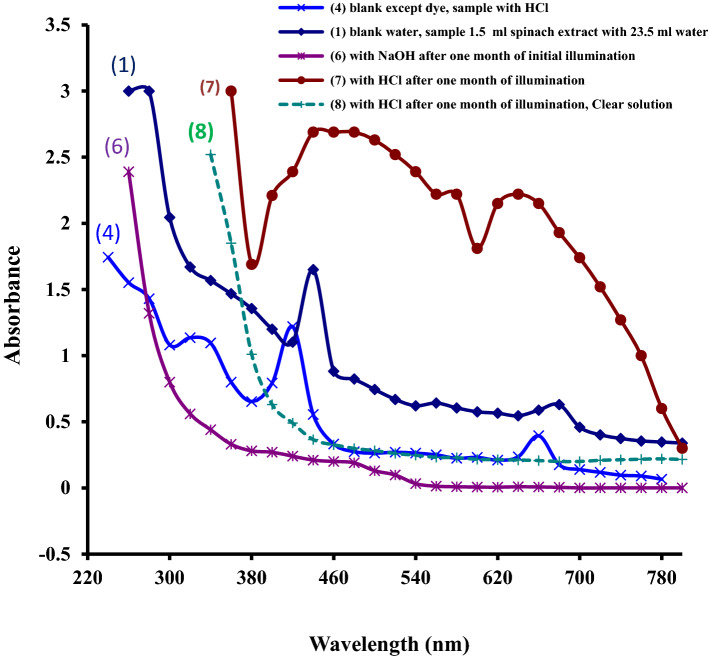
(1) Post-second time illumination spectra of the extract in the presence of NaOH, NaLS and Fructose [curve line 6,  main spectral property: both reference and sample solutions were illuminated on 2nd day of preparation, and re-illuminated on 24th day, spectra after re-illumination, no peak at any wavelength, A_0_ at 700 nm and higher λ, A_≈0_ from 540 to 700 nm, absorbance rises regularly from 540 nm towards lower λ]; (2) Post-illumination spectra of the extract in the presence of HCl, NaLS and Fructose. [curve line 7, main spectral property: absorbance at all λ was highly elevated, broad band at 640 nm (A_2.22_),very broad band at 440–480 nm (A_2.69_), a shoulder band at 540 nm (A_2.22_)]; and (3) Post-illumination spectra of the spinach extract in the presence of HCl, NaLS and Fructose: spectra of clear and transparent layer [curve line 8,  main spectral property: no peak at any λ, A_0_ at 700 nm and higher λ, A_≈0_ from 540 to 700 nm, absorbance rises regularly from 540 nm towards lower λ].

The spectra of crude spinach extract (present with NaOH, NaLS, Fructose in photogalvanic cell) after nearly 1 month and two times illumination, shows zero absorbance at 700 nm and higher wavelengths (Fig. 3, curve line 6) suggesting absence of turbidity and suspended matter. Absorbance from 540 to 700 nm is also negligible suggesting absence of chlorophyll, phaeophtin, etc. in the so old and illuminated photogalvanic electrolyte solution. It means that these molecules have been irreversibly photo-decomposed (obeying first order kinetics) by UV and visible light into unknown simpler molecules^64–76^. When this photo-degraded solution is again illuminated, the power output obtained is nearly equal to that for first time illuminated fresh crude spinach extract having NaOH, NaLS and Fructose (Table 1, Supplementary Table S2). It means that the fresh crude spinach extract as well as the photo-degraded extract containing NaOH are almost equally capable of power generation. It means that there is nothing to worry about photo-degradation of chlorophyll as far as power generation and storage is concerned through photogalvanics.

**Table 1:** Results of preliminary study for stability, storage capacity, and recharging of the crude spinach based cell over 23 days’ time (experiment no. 1).

Time duration^a^ (h)	Observations^b^ (current in mA cm^−2^)	Time duration^a^ (h)	Observations^b^ (current in mA cm^−2^)
0	i_max_ 56.25, i_sc_ 50.62	406.5	i_max_ 35.62, i_sc_ 18.75
46.5	i_max_ 47.50, i_sc_ 39.37	478.5	i_max_ 39.37, i_sc_ 22.50
166.5	i_max_ 45.00, i_sc_ 24.37	526.5	i_max_ 20.62, i_sc_ 17.81
262.5	i_max_ 46.87, i_sc_ 27.18	550.5	i_max_ 15.00, i_sc_ 10.78
358.5	i_max_ 39.37, i_sc_ 24.37	550.5^c^	i_max_ 43.75, i_sc_ 33.75
382.5	i_max_ 46.87, i_sc_ 26.25	–	–

^a^Throughout the study, the Pt and SCE were same. Time duration starting from initial charging by 1st time illumination in natural sunlight for 20 min of a newly fabricated cell. Time duration is not regular, and no observations for power at different times were made as such study was not planned for this manuscript. These observations were noted only out of curiosity to have preliminary idea about stability, storage capacity and recharging prospects of these devices.

^b^Circuit was kept open from 0 to 550.5 h in absence of illumination, and circuit was closed only when observations were noted at various time intervals.

^c^Cell was recharged at 550.5 h by illuminating for 30 min, and then observation was taken. P_pp_ (mW cm^−2^) after this recharge was 11.92, and at initial charging, P_pp_ was 13.75.

Therefore, it may be concluded that the crude spinach extract is good source for power generation without worrying about the stability/instability of chlorophyll due to NaOH or illumination. Although, it is the observed and reported fact that the stability and colour of chlorophyll is affected by the heat, air (oxygen gas), UV light and pH (Sect. 8 of SI).

The pre-illumination spectra of pure crude spinach extract (Fig. 1, curve line 1) and crude spinach extract (having HCl, NaLS and Fructose) (Fig. 1, curve line 4) have relatively sharp and low intensity bands and lower absorbance throughout the UV–visible region.

### Post-illumination spectra of crude spinach extract in highly acidic medium (pH 0.29) in presence of NaLS and Fructose (spectra of 33 days old illuminated electrolyte)

The post-illumination spectra of crude spinach extract in highly acidic medium (pH 0.29) in presence of NaLS and Fructose was also determined and compared with the pre-illumination spectra of the same solution. The crude spinach extract (pH 0.29) along with NaLS, Fructose and HCl was filled in photogalvanic cell and illuminated on 2nd day, and this already illuminated solution was again illuminated on 24th day, and then its UV–visible spectrum was taken on 33rd day.

The spectra of crude spinach extract (with HCl, NaLS, and Fructose) after nearly 1 month and two times illumination shows highly elevated absorption at 640 nm (A_2.22_, broad band), 580 nm (A_2.22_, shoulder band), 460 nm (A_2.69_, very broad band), and at all other wavelengths throughout the UV–visible region suggesting presence of excessive turbidity and suspended matter (Fig. 3, curve line 7; Supplementary Fig. S1; Supplementary Table S1). The solution was highly turbid due to the presence of yellow–brown waxy material which may be Phaeophytin formed by removal of Mg from chlorophyll. The observed bands may be attributed to the phaeophytin (in highly acidic medium in the presence of NaLS and Fructose). Broadness may be due to its peripheral Mg complex^77^.

When this highly turbid solution was left undisturbed for some time, then two layers were visible clearly. Upper layer was highly viscous, turbid, waxy and yellow–brown coloured whereas the lower layer was clear and transparent. The UV–visible spectra of this clear and transparent layer were found entirely different shown by curve line 8 in Fig. 3 (also see Supplementary Fig. S1, Supplementary Table S1) than that for the solution having both the layers mixed (shown by curve line 7 in Fig. 3). The spectra of this clear and transparent layer have no peaks, and resembles to that for 1 month old and two times illuminated alkaline crude spinach extract (shown by curve line 6 and 8 in Fig. 3) except the magnitude of absorbance. The value of absorbance for the transparent layer is higher than that for the later. As well the transparent layer has absorption above 700 nm showing presence of still some turbidity in clear layer. This turbidity may have entered the clear layer while separating it from upper viscous layer. If we make deduction of turbidity absorption (nearly 0.22) from spectra of clear solution, then spectral curve of this clear solution will appear to be overlapping with that for 1 month old and two times illuminated alkaline crude spinach extract for most part of the UV–visible region. It suggests that the chemical composition of clear acidic solution and 1 month old and two times illuminated alkaline crude spinach extract is nearly same. It means products of photo-decomposition in clear acidic solution and 1 month old and two times illuminated alkaline crude spinach extract is nearly same. It is suggestive of absence of chlorophyll and phaeophytin in clear solution as there is no peak in visible region.

Further, it is to be mentioned that upper waxy layer consists of water-insoluble organic substance (may be phaeophytin left-out from photo-damage). It seems that there was very high pheophytinization by NaLS at low pH (i.e., 0.29) which was reinforced by longer time (33 days). This led to the formation of highly viscous-turbid waxy material. This turbidity might have protected phaeophytin by causing light scattering and lowering photo-absorption by phaeophytin leading to lower photo-damage of phaeophytin in viscous part. This clear part was still clear on 48th day suggesting that pheophytinization was complete on 33rd day or before. Had it not been complete, some waxy matter would have been observed on 33rd day in clear part.

## Mechanistic aspect of the photo-generation of the current in the photogalvanic cells

A photogalvanic cell consists of two electrodes (say, platinum anodic and saturated calomel cathodic electrodes) in liquid electrolyte. Photo-stability and re-chargeability of photogalvanic solar cells is characteristics of its electrodes and electrolyte (Sect. 9 of SI).

The photogalvanic electrolyte is characterised by ionic species of sensitizer (chlorophyll). In addition to this, other ionic species/incipient ionic species like oxidised state of reductant molecule, alkali molecule, cationic/anionic surfactants’ molecule, photo-degradation products of sensitizer molecule, photo-excited and photo-reduced photo-degradation products of sensitizer molecule, etc. are also the chemical constituents of the electrolyte. Other ionic species/incipient ionic species are also the potential candidates for completing the internal circuit of cell leading to the current generation. This fact is the key for explaining the current generation from the old and used electrolyte in long term. The formation of ionic species from photo-reduction of chlorophyll is supported by published literature^59,78,79^.

## Photo-stability and re-chargeability of crude spinach extract based PG devices with reference to electrical output

As far as stability (with reference to electrical output) of crude spinach extract based PG devices is concerned, these devices have tremendous capacity of storing power for long time, and tremendous capacity of undergoing cycles of charging-discharging. Storage capacity and recharging prospects of crude spinach extract based photogalvanic cell has been studied for over 3 months and 20 days time (Table 1, Supplementary Table S2). Over this period, recharging with same and unclean Pt has been observed to show current up to 62–112% of current generated in initial charging. And, recharging with same and cleaned Pt has been observed to show current nearly equal to that generated in initial charging. Generation of nearly 86% and 55% of initial power (generated in initial charging) has been observed in recharging after 23 and 46 days, respectively. The deposition of material (removable) on Pt electrode reduces power output to some extent. Therefore, we view that use of same photogalvanic solution (NaOH, NaLS, crude spinach extract, and fructose) with same Pt and SCE after cleaning has potential of repeating performance observed in initial charging. Although for this view, an extensive study over longer period is needed (that shall be done by us in future), and which could not be part of this manuscript as it was not the aim of present study. I have observed similar and repetitive results by charging similar PG cells on different days in natural sunlight. The experimental work reported in this paper was done in year 2010. This work’s results have been reproduced many times under similar conditions of concentration etc. First time in year 2012, second time in year 2013 while getting cell verified from experts, and third time in year 2014 (Supplementary Table S3).

After recharge, the electrical performance is nearly same as in first time charging of the cell (provided the well cleaned Pt electrode is used at recharge). It gives inference that chemicals like reductant are not consumed otherwise nearly same power would not have been produced after recharging. Further, it also shows the photo-stability of crude spinach extract after its illumination and re-illumination. Had spinach extract not been stable, the same power output production on recharging would not have been possible.

Re-illumination of the cell is capable of reproducing result (potential and current) obtained by first time illumination (Table 1, Supplementary Tables S2, S3). It could not have been possible had the reductant been sacrificial. These results suggest that the main photochemical reaction is reversible, although some element of irreversibility cannot be denied as all natural processes are irreversible.

The recharging capacity of the cell to an electrical output value (value obtained after first time charging) shows that the PG electrolyte is not consumed otherwise electrical output value could not have been observed (Table 1, Supplementary Tables S2, S3). It shows that there is no substantial consumption of the electrodes, photo-sensitizer, reductant and surfactant during charging and discharging of the cell. It is estimated that there is only electron exchange between the electro-active species and electrodes, and photo-sensitizer molecules and reductant molecules in the bulk solution during photo-generation of current and power through the PG cells. Therefore, the PG cell studied in the present work is a photogalvanic system according the concept of photogalvanic effect laid down by the scientist *Rabinowitch*.

The main aim of the PG cells is power generation and storage. The present study shows that quite less costly and easy to fabricate spinach extract-based PG devices are quite stable *vis-à-vis* the solid-state cell devices based on the isolated photosynthetic protein-molecular complexes^80^ (Sect. 10 of SI).

## Conclusion

The spectra of crude spinach extract resembles to absorbance property of chlorophyll ‘a’ and chlorophyll–protein complex. Therefore, it may be concluded that chlorophyll–protein complex is the main component in crude spinach extract responsible for light absorption in visible region.

The high pH is not expected to adversely affect the physiological activity of crude spinach extract through pheophytinization as far as photogalvanics is concerned.

The spectra of crude spinach extract (present with NaOH, NaLS, and Fructose in photogalvanic cell) after nearly 1 month and two times illumination shows absence of thechlorophyll, phaeophtin, etc. in the so old and illuminated photogalvanic electrolyte solution. It means that these molecules have been irreversibly photo-decomposed. When this photo-degraded solution is again illuminated, the power output obtained is nearly equal to that for first time illuminated fresh crude spinach extract having NaOH, NaLS and Fructose. It means that the fresh crude spinach extract as well as the photo-degraded extract containing NaOH are almost equally capable of power generation. Therefore, it may be concluded that the crude spinach extract is good source for power generation without worrying about the stability/instability of chlorophyll.

The spectra of crude spinach extract (with HCl, NaLS, and Fructose) after nearly 1 month and two times illumination shows highly elevated absorption at all wavelengths throughout the UV–visible region suggesting presence of excessive turbidity and suspended matter. The spectra of clear and transparent layer existing over turbidity in the presence of HCl have no peaks, and resembles to that for 1 month old and two times illuminated alkaline crude spinach extract except the magnitude of absorbance. It suggests that the chemical composition of clear acidic solution and 1 month old and two times illuminated alkaline crude spinach extract is nearly same. It means products of photo-decomposition in clear acidic solution and 1 month old and two times illuminated alkaline crude spinach extract is nearly same. It is suggestive of absence of chlorophyll and phaeophytin in clear solution as there is no peak in visible region. As far as stability (with reference to electrical output) of crude spinach extract based PG devices is concerned, these devices have tremendous capacity of storing power for long time, and tremendous capacity of undergoing cycles of charging-discharging.

## Supplementary Information

Supplementary Information.
