# Supplementary material for: Characterization, stability, and feasibility of long-term use of light-absorbing components of aqueous spinach extract-based photogalvanic electrolyte
Source: Sci Rep. 2022 Aug 7;12:13518. doi: 10.1038/s41598-022-17647-5 (PMC9357696; doi:10.1038/s41598-022-17647-5)
Supplement: Supplementary file 1 — Supplementary Information. [file 41598_2022_17647_MOESM1_ESM.doc]

***Supplementary Information for***

**Characterization, stability, and feasibility of long-term use of light-absorbing components of aqueous spinach extract-based photogalvanic electrolyte**

Pooran Koli, Department of Chemistry, Jai Narain Vyas University, Jodhpur–342001, Rajasthan, INDIA; *poorankoli@yahoo.com, Tel. & Fax +91 291 2614162*

**1.** The edible portion (87 %) of Spinach (*Spinacia olerace*) contains (%) - moisture (92.1), protein (2.0), fat (0.7), fiber (0.6), mineral matter (1.7), carbohydrate (2.9) and oxalic acid (658 mg per 100 g). Mineral composition includes (mg per 100g)-Ca(73),Mg(84), Fe (10.9),P(21), K (206), Na (58.5),Cu (0.01), S (30), Ni (0.42), Mn (9.61), Mo(0.08), Zn (13.53) and Sr (0.077). In addition it contains selenium,vitamins (A,B1,B2,B3,B6,C,E,K), flavonoids, carotenoids (lutein, β-carotene, and zeaxanthin), folate, [omega-3 fatty acids](http://www.copperwiki.org/index.php?title=Omega-3_fatty_acid&action=edit&redlink=1), p-Coumaric acid, ascorbic acid, chlorophylls, and other substances ([*Nutrition and Cancer*,](http://www.ncbi.nlm.nih.gov/pubmed/14690799) 46, 222-231,2003).

**2.** Some other chemical components [*e.g., carotenoids (λ_max_ near 450 nm), Vt.B2, Vt.B12*] of the electrolyte (having the crude spinach extract) may also be playing role in the light absorption augmenting the absorption of the visible light by the chlorophyll-protein complex chromophore**^17^**. The electrolyte based on crude spinach extract is able to absorb the UV radiation**^30^,** although chlorophylls are not good absorber of the UV radiation**^30^**. It is evident from the fact that no absorption below 380 nm is reported for the chlorophylls. Therefore, the absorption in the UV region by the electrolyte may be attributed to the some chemical components (other than Vt.B_2_, carotenoids, and chlorophyll-protein complexes) present in the crude spinach extract. The crude spinach extract shows light absorption throughout the UV-Visible region. In the crude spinach extract, the chlorophyll ‘b’ and carotenoids including other accessory pigments are thought to transfer absorbed energy to the *chlorophyll ‘a’ molecule***^31-33^**. This inference of the energy transfer is derived from the fact that the light trapped by the accessory pigments helps the photosynthetic process. The chemicals like carotenoids and phycobilins are reported to sensitize the photosynthesis**^34^**. The photo-induced singlet energy of the accessory pigments is transferred as such to the chlorophyll molecule, and it may be attributed to the similarity of the excitation fluorescence spectra of the accessory pigment molecules and chlorophyll molecules**^31^**. The energy transfer efficiency from *chlorophyll ‘b’* *molecule* to *chlorophyll ‘a’ molecule* and *the phycobilins* *molecule* to *chlorophyll ‘a’* *molecule* is reported as approaching 100 %**^31^**. The energy transfer efficiency from the *carotenoids* *molecule* to the *chlorophyll ‘a’* *molecule* and *carotenoids* *molecule* to the chlorophyll ‘b’ *molecule* is reported about 30 % -70 %.

The chlorophyll-protein complex present in the crude extract is the main component harvesting the light. Therefore, the chemical and physical properties of the chlorophyll-protein complex are of very importance for the solar power generation and storage. In chlorophyll, the central magnesium coordinates four nitrogen atoms of the pyrrole rings, providing for one or both Mg axial positions to be occupied by a molecule possessing a lone electron pair capable of acting as an electron donor. Chlorophyll may also act as electron donor *via* its ring E and keto groups present in ring and side chains**^35^** (**Fig.S2**). Therefore, author view that intactness of Mg with chlorophyll facilitates electron transfer from reductant to chlorophyll and in turn from chlorophyll to Pt electrode used as anodic contact terminal in the photogalvanic cells. On removal of Mg, this property is lost and efficiency of cell is reduced. If a mixture of *chlorophyll ‘a’ and chlorophyll ‘b’* is excited with light absorbed mainly by *chlorophyll ‘b’*, and the pigment concentration is sufficiently high, the fluorescence will be emitted mainly by *chlorophyll ‘a’*. Because of the positions of the absorption and fluorescence maxima, the energy will be transferred mainly from chlorophyll ‘b’ to chlorophyll ‘a’, while the reverse is improbable. Chlorophyll can receive energy from some other excited molecules, and energy transfer from chlorophyll to chlorophyll is also possible in concentrated solution.

A different mechanism for energy transfer can occur if the concentration is so high that the average distance between the molecules is of the order of the molecular dimensions. In this case, the pie-electron clouds may overlap to such a degree that an electron can migrate easily from the excited state of one pigment molecule to another, while an electron of the ground state may move from the second molecule to the first. In solution, however, aggregation probably occurs before such high concentration is reached. Within such aggregates, the solid state phenomenon can occur, but the concentration is reduced so that between the aggregates no electron transport occurs. At all concentrations, energy transfer can occur by secondary fluorescence. Light emitted by one molecule is absorbed by another, as absorption and fluorescence spectra overlap partly. This process is inefficient, but it can interfere with measurements of fluorescence polarization and fluorescence quantum yields.

The life time of triplet state of *chlorophyll ‘a’* is 3 x 10^-3^ seconds, and depends strongly on pigment concentration**^32^**. Chloroplasts may be broken up without the use of detergents by overnight storage of a crude suspension of chloroplast fragments in the dark at 25 ^0^C. This results in the formation of chlorophyll-protein or pheophytin-protein complexes with red absorption maxima at 669 nm, which appears to be formed from chlorophyll ‘a’ at 683 nm after illumination. The absorption band of the *chlorophyll ‘b’* not changes**^24^**.

**3.** Composite spectral information including composition of reference (blank) solution and sample solution taken in reference cell cuvette and sample cell cuvette respectively-

1. **
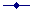
** Spectra of pure aqueous spinach extract (3 days old). Reference cell (singly distilled water), Sample cell **(**1.5 ml pure aqueous spinach extract + 23.5 ml singly distilled water, total 25 ml); Absorbance (*A*) slight but continuous increasing towards shorter wavelengths (λ), bands at 680 nm (A_0.63_), 440 nm (A_1.65_);
2. **
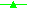
** Effect of NaLS (fresh) and Fructose (fresh) on the spectra of aqueous spinach extract (fresh). Reference cell (6ml Fructose, 4ml NaLS, 15 ml singly distilled water, total 25 ml); Sample cell **(**6 ml Fructose, 4 ml M/10 NaLS, 1.5 ml aqueous spinach extract, 13.5 ml singly distilled water, and total 25 ml); spectra taken after one hours of preparation of reference & sample solution. Absorbance slight but continuous increasing towards shorter λ, bands at 680 nm (A_0.36_), 420 nm (A_0.94_),340 nm (A_1.22_), shoulder band at 480 nm (A_0.55_).
3. **
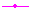
** Effect of NaOH (fresh) on the spectra of extract (fresh) having NaLS (fresh) and Fructose (fresh). Reference cell (12 ml NaOH, 6 ml Fructose, 4ml NaLS,3 ml singly distilled water, total 25 ml); Sample cell **(**12 ml NaOH, 6 ml Fructose, 4 ml NaLS,1.5 ml extract,1.5 ml singly distilled water, total 25 ml); spectra taken after 4 hours of preparation of reference & sample solution. Absorbance slight but continuous increasing towards shorter λ,bands at 680 nm (A_0.36_), 420 nm (A_0.94_), 340 nm (A_1.22_), shoulder band at 480 nm (A_0.55_).
4. **
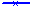
** Effect of HCl (7 days old) on the spectra of extract (5 days old) having NaLS (12 days old) and Fructose (5 days old). Reference cell (18 ml HCl, 9 ml Fructose, 6 ml NaLS, 2 ml singly distilled water, total 35 ml); Sample cell **(**18 ml HCl, 9 ml Fructose, 6 ml NaLS, 2 ml extract, 0.0 ml singly distilled water, total 35 ml); spectra taken after 29 hours of preparation of reference & sample solutions. Absorbance slight but continuous increasing towards shorter λ,bands at 660 nm (A_0.39_), 420 nm (A_1.22_), 320 nm (A_1.13_), shoulder band at 280 nm (A_1.43_).
5.
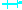
Effect of illumination on the spectra of extract (fresh) in the presence of NaOH (fresh), NaLS (fresh) and Fructose (fresh). Reference & Sample solutions as for spectra (c). Solutions were illuminated for 75 minutes after 4 hours of preparation of reference & sample solutions. A spectrum was taken immediately after illumination. Absorbance slight but continuous increasing towards shorter λ, broad band at 420 nm (A_0.75_), very broad band 640 nm (A_0.122_), shoulder band at 480 nm (A_0.37_).
6.
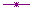
Post-illumination spectra of the extract (5 days old) in the presence of NaOH (7 days old), NaLS (12 days old) and Fructose (5 days old). Reference cell (18 ml NaOH, 9 ml Fructose, 6 ml NaLS, 2 ml water, total 35 ml); Sample cell **(**18 ml NaOH, 9 ml Fructose, 6 ml NaLS, 2 ml extract, 0.0 ml water, total 35 ml); Both reference and sample solutions were illuminated on 2^nd^ day of preparation, and re-illuminated on 24^th^ day, and then its UV-Visible spectra was taken on 33^rd^ day. No peak at any λ, A_0_ at 700 nm and higher λ, A_≈0_ from 540 nm to 700 nm, absorbance rises regularly from 540 nm towards lower λ.
7. **
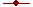
** Post-illumination spectra of the extract (5 days old) in the presence of HCl (7 days old), NaLS (12 days old) and Fructose (5 days old). Reference cell (18 ml HCl, 9 ml Fructose, 6 ml NaLS, 2 ml water, total 35 ml); Sample cell **(**18 ml HCl, 9 ml Fructose, 6 ml NaLS, 2 ml extract, 0.0 ml singly distilled water, total 35 ml); Both reference and sample solutions were illuminated on 2^nd^ day of preparation, and re-illuminated on 24^th^ day, and then its (sample solution became highly turbid & yellow-brown waxy) UV-Visible spectra was taken on 33^rd^ day. Absorbance at all λ was highly elevated, broad band at 640 nm (A_2.22_),very broad band at 440-480 nm (A_2.69_), a shoulder band at 540 nm (A_2.22_).
8. **
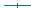
**Spectra of clear & transparent solution obtained from highly turbid & yellow-brown waxy sample solution described in spectra 6. Leaving turbid & waxy solution un-disturbed for some time led to appearance of two layers-waxy upper layer & clear-transparent lower layer. The spectra of this clear-transparent lower layer were taken. No peak at any λ, A_≈0.21_ at 600 to 800 nm, absorbance rises regularly from 600 nm towards lower λ.


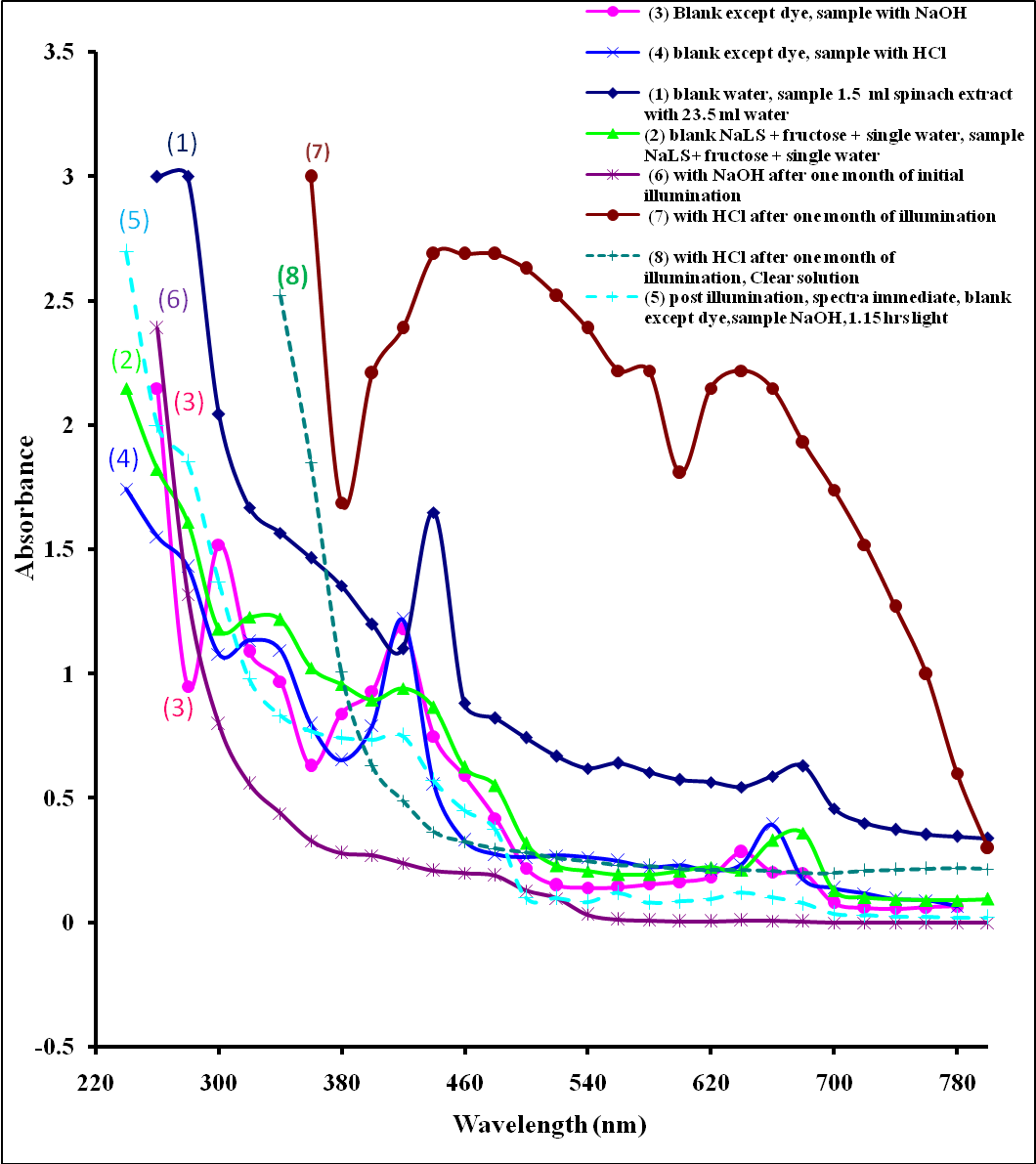


**Fig. S1:** **Composite UV-Visible spectra (see table S-1 for absorbance at individual wavelengths).**


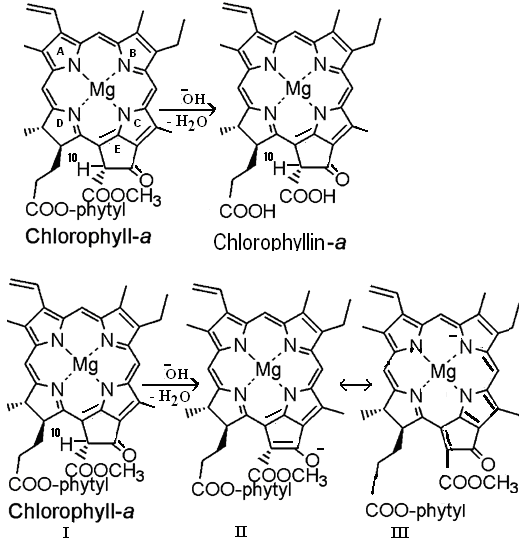


**Fig.S2:** Reactions of alkali with *chlorophyll ‘a’.*


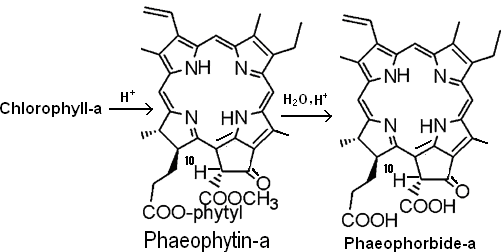


**Fig.S3:** Reactions of acid with *chlorophyll ‘a’.*

**Table. S1:** Absorbance at individual wavelengths for various samples

| **Wavelength (nm)** | **Absorbance values for** | | | | | | | |
| --- | --- | --- | --- | --- | --- | --- | --- | --- |
|  | *Spectra (1)* | *Spectra (2)* | *Spectra (3)* | *Spectra (4)* | *Spectra (5)* | *Spectra (6)* | *Spectra (7)* | *Spectra (8)* |
| 240 | **-** | 2.15 | **-** | 1.744 | 2.698 | **-** | **-** | **-** |
| 260 | 3.0 | 1.823 | 2.15 | 1.552 | 2.0 | 2.39 | **-** | **-** |
| 280 | 3.0 | 1.61 | 0.95 | 1.431 | 1.853 | 1.32 | **-** | **-** |
| 300 | 2.045 | 1.18 | 1.52 | 1.08 | 1.37 | 0.8 | **-** | **-** |
| 320 | 1.67 | 1.229 | 1.09 | 1.136 | 0.98 | 0.56 | **-** | **-** |
| 340 | 1.568 | 1.22 | 0.97 | 1.096 | 0.832 | 0.44 | **-** | 2.52 |
| 360 | 1.468 | 1.026 | 0.63 | 0.8 | 0.769 | 0.33 | 3.0 | 1.85 |
| 380 | 1.356 | 0.958 | 0.84 | 0.653 | 0.744 | 0.28 | 1.69 | 1.01 |
| 400 | 1.20 | 0.896 | 0.93 | 0.793 | 0.737 | 0.27 | 2.21 | 0.63 |
| 420 | 1.102 | 0.943 | 1.18 | 1.22 | 0.752 | 0.24 | 2.39 | 0.49 |
| 440 | 1.65 | 0.866 | 0.75 | 0.555 | 0.571 | 0.21 | 2.69 | 0.365 |
| 460 | 0.882 | 0.627 | 0.59 | 0.332 | 0.452 | 0.2 | 2.69 | 0.326 |
| 480 | 0.823 | 0.552 | 0.42 | 0.274 | 0.375 | 0.19 | 2.69 | 0.298 |
| 500 | 0.744 | 0.32 | 0.22 | 0.263 | 0.10 | 0.13 | 2.63 | 0.282 |
| 520 | 0.669 | 0.229 | 0.15 | 0.27 | 0.099 | 0.098 | 2.52 | 0.26 |
| 540 | 0.621 | 0.208 | 0.14 | 0.263 | 0.084 | 0.032 | 2.39 | 0.247 |
| 560 | 0.642 | 0.194 | 0.143 | 0.25 | 0.12 | 0.013 | 2.22 | 0.231 |
| 580 | 0.605 | 0.195 | 0.154 | 0.224 | 0.081 | 0.0087 | 2.22 | 0.229 |
| 600 | 0.575 | 0.208 | 0.164 | 0.231 | 0.085 | 0.0063 | 1.81 | 0.216 |
| 620 | 0.565 | 0.224 | 0.185 | 0.21 | 0.095 | 0.0056 | 2.15 | 0.214 |
| 640 | 0.545 | 0.211 | 0.284 | 0.235 | 0.122 | 0.0087 | 2.22 | 0.211 |
| 660 | 0.588 | 0.331 | 0.203 | 0.395 | 0.101 | 0.0074 | 2.15 | 0.207 |
| 680 | 0.63 | 0.361 | 0.2 | 0.175 | 0.078 | 0.0047 | 1.93 | 0.20 |
| 700 | 0.459 | 0.129 | 0.08 | 0.139 | 0.037 | 0 | 1.74 | 0.20 |
| 720 | 0.401 | 0.101 | 0.061 | 0.118 | 0.03 | 0 | 1.52 | 0.209 |
| 740 | 0.374 | 0.094 | 0.056 | 0.096 | 0.026 | 0 | 1.27 | 0.213 |
| 760 | 0.355 | 0.09 | 0.062 | 0.09 | 0.024 | 0 | 1.0 | 0.217 |
| 780 | 0.347 | 0.09 | 0.066 | 0.066 | 0.021 | 0 | 0.6 | 0.22 |
| 800 | 0.339 | 0.096 | **-** | **-** | 0.021 | 0 | 0.3 | 0.215 |
| 820 | - | **-** | **-** | **-** | 0.0367 | - | **-** | **-** |
| 840 | - | **-** | **-** | **-** | 0.0669 | - | **-** | **-** |

**4.** This change in absorption may be due to reasons**^25,29,36-38^** as described below-

**First,** A strong chlorophyll-chlorophyll interaction found in the complex is removed by ionic detergent NaLS. In the presence of NaLS, the chlorophyll-protein compounds of the alkaline spinach extract are dispersed yielding brilliantly clear solution. The chlorophylls as well as the carotenoids are probably oriented to the hydrophobic (paraffin) part of the NaLS molecule dissolving the chlorophylls and carotenoids. Because of this, the amount of turbidity and suspended particles is less resulting in lower absorbance throughout the spectrum. The absorbance at 750 nm and higher wavelengths shows that turbidity and suspended particles are still present in crude spinach extract.

**Two,** NaLS induces pheophytinization (eliminating of the magnesium from the chlorophyll) and conformational changes in the protein part of the complex leading to changes in the spectra. NaLS causes denaturing of the protein by altering the nature of the prosthetic group of the chlorophyll-protein compound (although the prosthetic group remains attached to the protein but the protein is split into smaller units). The chlorophyll or phaeophytin (depending on the pH) remains attached to the protein. The phaeophytin formation is reported inversely proportional to the pH. The NaLS enhances electrical performance of photogalvanic cells by increasing the solubility of chlorophyll –protein complex, but limitation is that it (NaLS) also causes removal of Mg from chlorophyll (called pheophytinization).The pheophytinization is low at high pH. Smith has reported that the pheophytinization reaction is extremely rapid in weakly acid solutions and takes place slowly in more alkaline solutions**^36^.** Therefore, the use of high pH favours the stability and performance of the extract to absorb light, although, the saponification of chlorophyll is expected to be high at high pH (**Fig.S2**).

These NaLS-induced changes of spectroscopic characteristics occurred in similar NaLS concentration ranges and were reversible**^38^**.

The aqueous solution of fructose on scanning in range 200 nm - 900 nm shows absorption in UV region (up to 340 nm) only with λ_max_ at 240 nm**^17^**. As far as fructose is concerned, no appreciable effect of reductant fructose is expected on spectrum as mild oxidizing and reducing agents have been reported to produce no observable change in the absorption spectrum of the chlorophyll-protein compound**^25^**.

Reductant fructose may also be playing role in stability and solubility of Chlorophyll ‘a’/’b’-Protein-​Komplex. Reductants are reported to favor solubility and stability of proteins by disfavouring their denaturation**^39^**.

It is reported that if there are molecules present that can act as oxidant or reductant, the energy of excitation may be expended in transferring an electron by reactions as below**^24^**-

Chl* + Ox → Chl^.+^ + Ox^.-^ ; Chl* + Red → Chl^.-^ + Red^.+^,

Chl + Ox → Chl^.+^ + Ox^.-^ ; Chl + Red → Chl^.-^ + Red^.+^

The reactions are much faster if not coupled with proton transfer. Another possible process is electron ejection as**^40^**, Chl + light → Chl^.+^ + e^-^ . According to *Krasnovskii and Drozdova*, the photo-reduction of chlorophyll is very effectively inhibited by carotene, lutein (ten), and violaxanthin (nine), less effectively inhibited by vitamin A acetate (five), a tetraene, and beta-ionone, and not inhibited by allocymene (2,4,6-octatriene) and crotonic acid. They attributed the inhibition to re-oxidation of reduced chlorophyll by the carotenoid, although they were unable to demonstrate rduction of any of the carotenoids. They also found inhibition by tetracene and anthracene**^41^**. On these facts, the author view that the reductant’s effect on chlorophyll spectra is negligible. Therefore, the combined effect of NaLS & fructose on chlorophyll spectra in present study may be attributed to mainly NaLS.

**5.** This change in absorption spectrum may be due to saponifying**^25^**, solubilizing**^25,42^** ,denaturizing**^25^**, and enolization**^43-45^** effect of NaOH on chlorophyll-protein complex**.** Chlorophyll is reactive towards a base yielding a series of phyllins, and magnesium porphyrin compounds.

The ester groups of chlorophylls of chlorophyll-protein complex undergo saponification in strongly alkaline medium (**Fig.S2).** The rate of saponification is reported to be a direct function of the hydroxyl ion concentration. Protein denaturation and the change in spectrum appear to be roughly parallel. In denaturation, it is to be mentioned that there is change in only secondary and tertiary structure of protein. Primary structure and porphyrin structure is reported to be intact**^25^**. Chlorophyll reacts with OH^-^ ions (source NaOH) to give the bright green chlorophyllin pigment. It is reported that the hydrolysis of both chlorophylls with cold dilute alkali (KOH) solution gives one molecule of phytol, one molecule of methanol, and one molecule of chlorophyllide ‘a’ or chlorophyllide ‘b’**^46^**.

Strong base (e.g. NaOH) abstracts the C-10 hydrogen of chlorophylls (**Fig.S2**) and pheophorbides, leaving a yellow to yellow-orange species (phase test intermediates) which is presumably the enolate ion. Under these conditions, the phase test intermediate is soon saponified. Weller has reported spectra of intermediates shows lower absorbance and broad peaks in UV-VIS spectra. The spectrum of chlorophyll ‘a’ in the absence of NaOH has higher absorbance and sharp peaks. The profound alteration in the spectrum on making the phase test intermediate suggests that its negative charge is not confined to the oxygen of the enolate ion but is distributed over the entire conjugated system**^24,43^**. In this structure, the conjugated system no longer makes a closed loop, and an altered spectrum might be expected**^47^**.

The absorbance throughout the 440 nm-800 nm regions (except near 640 nm) is reduced in comparison to that for crude spinach extract containing NaLS with fructose. It may be due to reduced turbidity as solubility of porphyrins (and their metal complexes) is reported to increase with increase in pH above 11**^42^**. Visible absorption, with a slight but continuous increase towards shorter wavelengths and absorption at 750 nm and higher wavelength suggest that even in NaOH, some turbidity and suspended particles are still present in extract.

In present study, this saponification proves to be blessing in disguise as it increases the solubility of chlorophyll-protein complex, and –COO^-^ resulting from saponification also facilitates electron transfer from chlorophyll to Pt electrode (**Fig.S2)**. As well the enolate anion formation will also do the same.

*Smith* has reported that at pH 9.0, the leaf extract is quite stable and shows no change in its solubility, precipitation properties, or spectrum. In M/10 alkali, the protein is gradually precipitated. With very strong alkali (5 M), a precipitate of denatured protein forms immediately. Smith has also reported that decrease in absorbance with time is low at high pH than low pH**^36^**. The solubility of porphyrins (and their metal complexes) is reported to increase with increase in pH above11 also**^42^.**

In present study, M/10 NaOH has been used, and resultant pH of the solution used in cell is of the order of 13.72. Despite such a higher pH use, no negative effect reported of the high pH is observed in present study. Instead of this, high pH has proved very useful by removing adverse effect of NaLS (pheophytinization) and enabling NaLS to have only positive effect of solubilising the chlorophyll-protein complex. The NaOH itself has solubilising effect on chlorophyll-protein complex, and also creates anionic chlorophyll structure enhancing the electron donating power of the extract. All this cumulative effect of NaOH enables the crude extract to show higher electrical performance in photogalvanic cell. At low pH (pH 0.29), the electrical output observed for cell is low and the crude spinach extract has also been found to undergo precipitation in present study. Contrary to this, at high pH (of the order of 13.71), the electrical output observed for cell is very high and the crude spinach extract has also been found stable *vis-a-vis* precipitation in present study. This way in present study, the high pH is not expected to adversely affect the physiological activity of crude spinach extract as far as photogalvanics is concerned. The pH of the solution used in cell does not remain constant as buffer has not been used in present study. At the pH of the order of 13.71, no precipitation has been observed in solution even after very long time. It is suggestive of physical stability of crude spinach extract in cell.

Higher pH is must in this cell as at lower pH the potential is very low (500 mV), and therefore, power is also very low (at low pH (0.29), i_sc_ 27.18 mAcm^-2^,V_max_ 0.460 V, V_oc_ 0.453,P_pp_ 4.18 mWcm^-2^;and at high pH, i_sc_ 50.62 mAcm^-2^, V_max_ 1.03 V, V_oc_ 0.996, P_pp_ 13.75 mWcm^-2^). In addition to poor cell performance, very high pheophytinization, precipitation and extreme turbidity has been observed at low pH.

Smith has also reported that the pheophytinization reaction is extremely rapid in weakly acid solutions and takes place slowly in more alkaline solutions. This change is apparent by the striking colour change from the original brilliant green first to an olive green and finally to a yellow or brown. It should be emphasized that NaLS removes magnesium from the chlorophyll-protein compound in neutral and in slightly alkaline solutions. The visible spectrum of the alkaline solution measured immediately after addition of the NaLS shows no significant change in spectrum, if solubilising effect of NaLS is overlooked. It gives inference that pheophytinization is disfavoured in alkaline medium.

In present study, the high pH (13.71) has been used. Therefore, author estimate absence or insignificant amount of pheophytinization in crude spinach extracts.

In present study in given conditions (NaLS, Fructose), the precipitation of protein is not visible even at 13.71 pH and even after one month of mixing of NaOH with extract. It suggests that in present study, the chlorophyll-protein complex is quite stable. As well, the chlorophyll is stable except ester hydrolysis and enolization. The porphyrin ring with Mg seems to be intact in absence of illumination/or weak illumination. In short time, the porphyrin ring is thought to be intact after strong illumination.

The effect of the NaOH may be less due to the fact that 13.71 is initial pH when NaOH is mixed. After mixing, some NaOH is used for saponification and enolization of chlorophyll. So in the course of time, the effective pH is less than 13.71, and safe for chlorophyll-protein complex leading no precipitation. Further, no foul smell in solution containing crude spinach extract (a biomaterial) has been observed even after very long time. The absence of precipitation shows that there is no denaturation of chlorophyll-protein at pH of the order of 13.71, in present study.

The pH of the solution used in cell does not remain constant as buffer has not been used in present study. At the pH of the order of 13.71, no precipitation has been observed in solution even after very long time. It is suggestive of physical stability of crude spinach extract in cell.

Higher pH is must in this cell as at lower pH, the potential is very low (500 mV), and therefore, power is also very low (at low pH (0.29), i_sc_ 27.18 mAcm^-2^,V_max_ 0.460 V, V_oc_ 0.453, P_pp_ 4.18 mWcm^-2^; and at high pH, i_sc_ 50.62 mAcm^-2^, V_max_ 1.03 V, V_oc_ 0.996, P_pp_ 13.75 mWcm^-2^). In addition to poor cell performance, very high pheophytinization, precipitation and extreme turbidity has been observed at low pH.

In weakly acid solutions, magnesium is rapidly eliminated from the chlorophyll, converting it to phaeophytin; in alkaline solutions, this reaction takes place very slowly. And at very high pH, the pheophytinization reaction is not detectable even after very long time, and prosthetic group (chlorophyll) remains attached to the protein component**^29^**. Therefore, in present study at high pH, detachment of prosthetic group (chlorophyll) from protein, and pheophytinization reaction is thought to be absent. It gives inference that chlorophyll-protein complex and in turn spinach extract remains physiologically intact in presence of such a high pH. At low pH (0.29), the electrical output observed for cell is low (i_sc_ 27.18 mAcm^-2^, V_max_ 0.460 V, V_oc_ 0.453, P_pp_ 4.18 mWcm^-2^), and the crude spinach extract has also been found to undergo precipitation at such a low pH. Contrary to this, at high pH (of the order of 13.71), the electrical output observed for cell is very high (i_sc_ 50.62 mAcm^-2^, V_max_ 1.03 V, V_oc_ 0.996, P_pp_ 13.75 mWcm^-2^), and the crude spinach extract has also been found stable *vis-a-vis* precipitation. This way, the high pH is not expected to adversely affect the physiological activity of crude spinach extract as far as photogalvanic is concerned. Bleaching increases strongly with increasing acidity**^8.^**

Metallo-porphyrins like chlorophylls are electrochemically active and their high stability allows redox reactions to proceed under severe conditions. The increased solubility of Metallo-porphyrins at higher pH favours their electrochemically active. At higher pH, reducing property of reductant is also high**^42^**.

The chlorophyll is hydrolyzed in acidic medium (**Fig.S3)** but anionic form of chlorophyll with –COO^-^ is not possible in acidic medium. Therefore, the electron donating tendency of *chlorophyll ‘a’* to Pt is thought to be reduced in acidic medium.

**6.** This change in absorption spectrum may be due to pheophytinization, denaturization, and hydrolysis of chlorophyll-protein complex in acidic medium. Strong acids modify the absorption spectrum by converting the chlorophyll-protein complex by pheophytinization of chlorophyll and denaturization of protein**^25^**. Phaeophytin ‘a’ is a bluish black waxy pigment. Phaeophytin ‘a’ and Phaeophytin ‘b’ is gray-brown and yellow-brown in colour, respectively. In the presence of NaLS, the removal of Mg of the chloroplast pigment is catalyzed by hydrogen ions. The removal of Mg is reported to be proportional to hydrogen ion concentration**^36^**. It is to be mentioned that there is immediate change in colour when HCl is mixed in crude spinach extract. It may be due to immediate pheophytinization. Hydrolysis of chlorophylls (reverse of esterification) splits off phytol and gives phaeophorbides (**Fig.S3**).

No anionic form of chlorophylls is possible in acid medium. Therefore, in acidic medium, the chlorophylls have less electron donating power to Pt electrode and in turn lower power generation than that for chlorophylls in strongly alkaline medium in photogalvanic cells.

In acidic medium, the lower electrical output for cell may also be due to Mg free porphyrins and the protonation of N or O atoms of chlorophyll-protein complex and Mg free porphyrins as the Mg free porphyrins and protonated structures have less electron donating tendency.

The photogalvanic devices reported in this manuscript uses crude spinach extract along with NaLS, NaOH (high pH), and Fructose. Therefore, the UV-Visible spectroscopic study of crude spinach extract was done to have knowledge about the photo-stability of extract in the photogalvanic cell.

**7.** A post-illumination spectrum of crude spinach extract having NaOH, NaLS and fructose is only slightly different than that for pre-illumination spectra of same photogalvanic solution. Reasons may be –

(a) Absence of organic solvents as aqueous medium has been used in present study. It is reported that chlorophyll is photo-labile in organic solvents and stable to high light intensities for long periods in the aqueous extracts**^25^**.

The radicals are reported to be responsible for photo-bleaching of chlorophylls**^35^**. In aqueous medium as in present study of crude spinach extract, water (which mainly absorbs wavelengths 167 nm, 125 nm) not absorbs in near UV region, so no excitation and no cationic radical formation of water takes place, that is needed for photo-bleaching of chlorophylls. Whereas the organic solvents like acetone (which mainly absorbs wavelengths 187 nm, 270 nm) absorbs strongly in near UV region to form acetone cationic radical needed for photo-bleaching of the chlorophyll like molecules. The bleaching of chlorophylls is claimed to be related to the absorption by the triplet state of chlorophyll (**^3^**Chl) in organic solvents**^50^**.

It is to be mentioned that sunlight have negligible amount of far UV radiations (< 200 nm) on earth surface as these are absorbed by stratosphere.

**(b)** Span of light exposure of crude spinach extract was short. Photo-bleaching depends on the intensity or time-span of light exposure**^35^**; it is reported that, unlike the one-electron oxidation of the chlorophyll, the reduction (of chlorophylls) is usually reversed immediately upon cessation of illumination, reduction of chlorophyll leads to stable non-radical products. There is evidence that the reduction is initiated by a one-electron step [Chl + Red → Chl^.-^ + Red^.+^]. *Evstigneev and Gavrilova* have reported that a gold electrode in a solution having ascorbic acid and chlorophyll became more negative on illumination, by about 0.3 volt. They regarded this as evidence for the presence of the reduced semiquinones of the pigments**^51-52^**.

In present study, the electrolyte containing the chlorophylls along with reductant and surfactant was illuminate for short and illuminating source was cut-off to bring electrolyte back in dark. In dark, author expects re-oxidation of reduced chlorophylls, and it is supported by study reported by scientists-*Krasnovskii, and Livingston.* Krasnovskii reported that Chlorophyll ‘a’ was photo-reduced by ascorbic acid in pyridine to a red compound (ChlH_2_), with an absorption band at 525 nm**^53^**. ChlH_2_ is re-oxidized to Chlorophyll in the dark by dehydroascorbic acid, O_2_, and such oxidants as Safranine T, Riboflavin, Fe^+3^, Quinine, Thionine, etc.**^24^**. *Livingston & Pugh* have observed that the chlorophyll can be photo-chemically reduced, and this Chlorophyll-sensitized *in-vitro* photo-reduction reaction occur through triplet state. The reaction is reversible. But, the regeneration of chlorophyll is never complete as part of it (chlorophyll) is converted into phaeophytin. There is little reduction in very dry pyridine; the reaction rate is considerably increased by the presence of at least 5 % water or alcohol**^54-55^**.

Reported photo-potential studies also lend support to view of reversibility element in photo-reduction of chlorophylls. The Chl/Chl^+^ half cell potentials are most reliably estimated by polarography. First presumably marking reaction, Chl → Chl^.+^ + e^-^ , has been reported by scientist *Stanienda* for chlorophyll and pheophytins in acetonitrile**^56^**. The scientist *Gilman has* reported determination of polarographic half-wave reduction potentials for a number of chlorophyll derivatives in ethanol, measured against an ethanolic saturated calomel electrode (potential +0.064 volt *vs* NHE) consisting of Hg/Hg_2_Cl_2_/Sat.NH_4_Cl in ethanol. The first two electron wave corresponds to the electrode reaction, Chl + 2e^-^ + 2 H^+^→ Chl + H_2_, but the potential E_1/2_ is by the reaction, Chl + e^-^ + H^+^→ Chl + H., Half wave potentials for several tetraphenylporphin derivatives have also been reported**^24,57^**. Similar cathodic potentials have been reported by Felton *et al.* for chlorophyll ‘a’ (-1.1 volt) and chlorophyll ‘b’ (-1.05 volt) in dimethylsulfoxide versus the aqueous S.C.E. electrode**^58^**. *Evstigneev and Gavrilova* have reported the change in the potential of a gold electrode immersed in the solution, brought about by photo-reduction of chlorophyll and pheophytins by ascorbic acid in pyridine**^59,60^**.

During photo-reduction of *Chlorophyll ‘a’*, *Chlorophyll ‘b’*, and pheophytin a+ b, the electrode potentials dropped from their dark values of -0.37, -0.34, and -0.36 volt (SCE), to -0.66, -0.59, and -0.70 volt, respectively. When the light was turned off, the dark values were slowly restored. The photo-potentials approximated to the equilibrium potentials for the couple Chl/ChlH_2_,-0.035 Volt determined by chemical means**^61^.** Reversible potential drop were also noted on illuminating *Chlorophyll ‘a’*, bacterio-chlorophyll, and chlorobium chlorophyll with Na_2_S in pyridine, but to lower values,-0.85, -0.90, -0.80 volt (SCE)**^62^**.

(c) Chlorophyll has robust electronic structure due to porphyrin ring;

(d) Reductant (fructose) may be favouring the stability of protein part of chlorophyll-protein complex**^39^**;

(e) The use of the crude extract may also be helping in using multiple roles of carotenoids like photo-protection via quenching of chlorophyll excited triplet states, and light harvesting via energy transfer to chlorophyll**^63^**.

Photo-damage of chlorophyll can arise from production of chemically reactive singlet oxygen because of the interaction of ground state triplet oxygen with the excited state chlorophyll triplet. β-Carotene can serve as an alternative acceptor of the excited state chlorophyll triplet, preventing the production of excited singlet oxygen**^24^.**

**8.** Although, it is the observed and reported fact that the stability and colour of chlorophyll is affected by the heat, air (oxygen gas), UV light and pH.

Chlorophylls are not efficient UV absorbers, but are still able to absorb UV radiation, especially around 350 nm. The irradiation of chlorophyll electrolyte solution with the UV and Visible light results in the irreversible breakdown of chlorophyll, accompanied by the appearance of a number of intermediate and final products**^30^**. The chemical structure of these products is largely unknown because of the diversity of the pathways involved and the lability of the primary photoproducts**^65^**. Photobleaching of chlorophyll in solution may proceed without pheophytinization and could involve the opening of the porphyrin ring**^66^**. Small amounts of allomers have also been detected after bleaching**^67^**. Photo-bleached chlorophyll is not identical with the triplet state, but is produced from it, because carotene and oxygen inhibit the reaction**^68^**.

The Chlorophyll ‘b’ and Mg phthalocyanine are photo-bleached to about the same extent as *chlorophyll ‘a’*, but pheophytin ‘a’, protoporphyrin, and mesoporphyrin are bleached to a much smaller extent or not at all. Electron transfer from the solvent to the porphyrin has been suggested as the cause of photobleaching**^69^**. It is difficult to reconcile this explanation with the fact that although the quantum yield is very low, it is practically independent of solvent**^68^**, and not enhanced in the presence of reductants**^70^**. Cu pheophytin and hemin are probably bleached by electron transfer from the ligand to the central metal. Singlet oxygen also attacks the double bonds of the phytyl chain leading to the formation of numerous isoprenoid photoproducts**^71^**.

Towards lower wavelengths from 540 nm, the absorbance rises continuously. This absorbance at lower wavelengths may be due to photo-degradation products of chlorophyll, and due to simpler molecules already present in crude spinach extract.

The reasons for irreversible photo-bleaching of chlorophyll after illuminations followed by long span of time may be –

1. Irreversible photo-bleaching may be there in photogalvanic cell electrolyte solution due to air in solution as reversible photo-bleaching is not possible in presence of oxygen (air)**^69^**.

*Porret and Rabinowitch* discovered that deoxygenated solutions of chlorophyll in methanol were perceptibly bleached when exposed to intense light**^72^**. *Livingston* *et al.* confirmed this reversible photo-bleaching or phototrophy of chlorophyll, and studied the reaction intensively**^68,69,73^**. The extent of bleaching is proportional to the square root of light intensity, and amounts to about 1 % at their highest light intensities. The recovery of chlorophyll in dark is complete in one second if the solvent is pure. The back reaction in methanol is second order in bleached product concentration**^73^**, but in viscous solvents tends towards first order**^68^**. The quantum yield is about 5 x 10^-4^ only**^73^**, but the extent of bleaching is enhanced by traces of oxalic acid or I_2_. Bleaching occurs in polar solvents, but not in dry benzene unless activated with about 1 % of methanol**^68^**.

Reversible bleaching at 650 and 680 nm has been ascribed to artifacts arising from light-induced fluorescence yield changes**^74^**.

In present study, the use of air free solution is neither necessary nor practicable as it will add to the cost of power production, and photo-degraded products have more or less same power output (**Table 1, Table S2, Table S3**).

(b) Spectra taken immediately after first time illumination shows similarity in spectra to pre-illumination spectra. It may be that carotene type molecules present in crude spinach extract mitigate the effect of photo-bleaching by helping in the stability of chlorophyll. But after many illuminations followed by long time passage, carotenes have no such ability may be by their own photo-decay.

(c) Ultraviolet radiation can induce the generation of free radicals in organic compounds, especially those containing C=C bonds. In chlorophylls, these bonds appear in great numbers**^75^** (**Fig.S2**). Once created, free radicals can initiate chain reactions, which, in some cases, may be linked particularly to the presence of C=C and C=O bonds**^35,76^**.

**9.** Photo-stability and re-chargeability of photogalvanic solar cells is characteristics of its electrodes and electrolyte The electrolyte consists of photosensitizer, reductant, surfactant and alkali in aqueous medium. The external circuit between electrodes is made of copper connecting wires, and internal circuit between electrodes is completed by diffusion of ions in electrolyte solution. Singly distilled water and un-ionic chemicals species of reductant and surfactant contributes negligible current. But, the ionic species contribute to the current of the cell. In the mechanism of the photo-generation of current from the photogalvanic cells, the photo-excited species of the photosensitizer undergoes reduction by the process of transfer of an electron from reductant molecule to sensitizer molecule. The sensitizer molecules have all electrons paired. The photo-excitation of sensitizer molecules leads to an electronic state having two unpaired electrons. Triplet excited electronic state is characterised by two unpaired electrons having parallel spins (di-radical structure). Singlet excited electronic state is characterised by two unpaired electrons having opposite spins (di-radical structure). One electron reduction of these excited electronic states of the sensitizer (as chlorophyll molecule in present study) leads to the formation of anionic radical structure (semi form). It is this anionic radical form which is an elctro-active species to give electron to platinum electrode in illuminated chamber of the photogalvanic cell. The formation of ionic species from photo-reduction of chlorophyll is also supported by published literature. Conductivity changes have been noted during reduction by phenylhydrazine in pyridine, indicating increase of ionic species during reduction of porphyrins**^59,78^**.

The determination of potential for reduction is reported by cyclic voltametery. A single, reversible, one electron reduction near 1 .5 volt *vs* SCE to lead to formation of anion radical, etc. is reported**^79^**.

**10.** The reproducibility of the phenomenon described in the manuscript has been checked in two ways-First, by comparing the results of the present study with already published work. The spectra of crude aqueous spinach extract have been found similar in present study to that for intact chloroplast, except in the UV region. The spectra of intact chloroplast have two absorption maxima at 678.5 nm (Q band; A_0.875_) and 437 nm (Soret band with A_1.8_)**^22-24^**; Second, by repeating the observations of the electrical output of the same electrolyte by recharging at different time. In present study, the results of different time are same or negligibly different. And, four experiments done one after another in total four years repeat the result (year 2014) **(Table S3).**

# Therefore, the present study is definitely presenting new insights towards advancing solar energy conversion technologies by showing the performance attributes of a plant-chromospheres based device. There is bit problem of evaporation of water from solution. This problem could be tackled by sealing of the cell. This aspect and use of crude extract of leaves and other parts of plant (also from waste of agriculture and forestry) is also a good point for future study.

**Table S2. Results of preliminary study for stability, storage capacity, and recharging of the crude spinach based cell (same cell) over three months & 20 days time (experiment no.2)**

| ***Time duration* (Hours)*** | ***Observations*****  ***(current in mAcm^-2^)*** | ***Time duration* (Hours)*** | ***Observations*****  ***(current in mAcm^-2^)*** |
| --- | --- | --- | --- |
| 0 | i_max_ 68.75, i_sc_ 50.00 | 1105.40 | i_max_ 38.75, i_sc_ 06.50 |
| 17.00 | i_max_ 66.25, i_sc_ 49.62 | 1105.40*** | i_max_ 40.00, i_sc_ 31.25 |
| 22.00*** | i_max_ 62.50, i_sc_56.25 | 1105.40**** | i_max_ 62.50, i_sc_ 49.54 |
| 25.08 | i_max_ 62.50, i_sc_ 43.75 | 1297.40 | i_max_ 42.50, i_sc_ 32.50 |
| 44.20*** | i_max_ 62.50, i_sc_ 50.00 | 1441.40 | i_max_ 37.50, i_sc_ 31.25 |
| 87.40 | i_max_ 56.25, i_sc_ 35.00 | 1441.40*** | i_max_ 62.50, i_sc_ 37.50 |
| 481.40 | i_max_ 45.00, i_sc_ 20.00 | 1537.40*** | i_max_ 62.50, i_sc_ 50.00 |
| 625.40 | i_max_ 17.00, i_sc_ 10.00 | 2065.40 | i_max_ 27.50, i_sc_ 25.00 |
| 769.40 | i_max_ 8.75, i_sc_ 8.75 | 2209.40*** | i_max_ 62.50, i_sc_ 37.50 |
| 1009.40 | i_max_ 6.50, i_sc_ 6.50 | 2641.40*** | i_max_ 50.00, i_sc_ 40.00 |

********Throughout the study, the Pt and SCE were same. Time duration starting from the initial charging by 1^st^ time illumination in natural sunlight for the 13 minutes of a newly fabricated cell. Time duration is not regular, and no observations for power at different times were made as such study was not planned for this manuscript. These observations were noted only out of curiosity to have preliminary idea about stability, storage capacity and recharging prospects of these devices.*

*********Circuit was kept open throughout the study from 0 to 2641.4 hours, and circuit was closed only when observations were noted at various time intervals. Circuit was also kept open while recharging by illumination. Throughout the study (from 0 to 2641.4 hours), the cell was un-illuminated, and cell was illuminated only at some time intervals for some time only and then illumination was cut-off.*

**********Cell was recharged at 22, 44.2, 1105.4, 1441.4, 1537.4, 2209.4, and 2641.4 hours by illuminating for 30 minute each time and then observations were taken. P_pp_ (mWcm^-2^) after recharge at 1105.4 and 2209.4 hours was 12.96 and 7.031 respectively, and at initial charging, P_pp_ was 13.05.*

***********Pt was cleaned and reused in already illuminate cell at 1105.4 hours for 30 minutes, and all future ward study was done with this cleaned Pt.*

***Table S3:*** *Results observed for a PG cell and its electrolyte at different time (years) in natural sunlight*

| **Year** | ***i_sc_** | **Current density**  ***w.r.t.* Pt 0.08 cm^2^** | **Power** | **Power density *w.r.t.* Pt 0.08 cm^2^** | **% Efficiency**  ***w.r.t.* Pt Area** |
| --- | --- | --- | --- | --- | --- |
| 2010 | 4000.0 µA | 50.00 mAcm^-2^ | 1044.0 µW | 13.05 mWcm^-2^ | 13.05 |
| 2012 | 4050.0 µA  4000.0 µA | 56.25 mAcm^-2^  50.00 mAcm^-2^ | 1100.0 µW  1044.0 µW | 13.75 mWcm^-2^  13.05 mWcm^-2^ | 13.75  13.05 |
| 2013 | 4050.0 µA  3980.0 µA | 56.25 mAcm^-2^  49.75 mAcm^-2^ | 1057.0 µW  1038.4 µW | 13.21 mWcm^-2^  12.97 mWcm^-2^ | 13.21  12.97 |
| 2014 | 3900.0 µA | 48.75 mAcm^-2^ | 1039.4 µW | 12.99 mWcm^-2^ | 12.99 |

# **At [Fructose] = 2.35 x 10^-3^ M, Crude spinach extract = 3.8 ml, Pt electrode area = 0.4 x 0.2 cm^2^, illuminated area of cell 1 x 1 cm^2^, natural sunlight intensity=100 mWcm^-2^, diffusion length (D_L_) = 6.3 cm, pH = 13.72.*
